# Association between selenium levels and oesophageal adenocarcinoma risk: evidence from a meta-analysis

**DOI:** 10.1042/BSR20160131

**Published:** 2016-07-08

**Authors:** Bin Hong, Lihong Huang, Ning Mao, Tao Xiong, Chao Li, Liangbo Hu, Ying Du

**Affiliations:** * Department of Thoracic Surgery, Yongchuan Hospital, Chongqing Medical University, Chongqing 402160, China; † Department of Ultrasound, Yongchuan Hospital, Chongqing Medical University, Chongqing 402160, China; ‡ Department of CT, Yongchuan Hospital, Chongqing Medical University, Chongqing 402160, China

**Keywords:** meta-analysis, oesophageal adenocarcinoma, selenium

## Abstract

Quantification of the association between selenium and risk of oesophageal adenocarcinoma (OAC) is still conflicting. The purpose of this meta-analysis is to explore the relationship between selenium levels and OAC risk. PubMed and Web of Knowledge were searched for the related articles. Pooled relative risks (RRs) with 95% confidence intervals (CIs) from random effects models were calculated. Sensitivity analysis and publication bias were conducted. Dose–response relationship was assessed by restricted cubic spline and variance-weighted least squares regression analysis. Five articles involving 748 OAC cases were included in this meta-analysis. Pooled results suggest that higher selenium level was not significantly associated with the risk of OAC (summary RRs=1.08, 95% CIs=0.84–1.39, *I*^2^=0%). Besides, no significant association was found in case-control studies (summary RRs=1.13, 95% CIs=0.84–1.52, *I*^2^=0%) or cohort studies (summary RRs=0.99, 95% CIs=0.55–1.78, *I*^2^=32.6%). A linear dose–response relationship was attested that an increase in dietary selenium intake of 10 μg/day is marginally associated with 1% increase in the risk of developing OAC (summary RRs=1.01, 95% CIs=0.99–1.03), but not statistically significant. No publication bias was found. In conclusion, our analysis indicated that a higher selenium level was not significantly associated with the risk of OAC. The relevant further studies are warranted.

## INTRODUCTION

Over the past several decades, the incidence of oesophageal adenocarcinoma (OAC) has risen more rapidly in many high-income countries for reasons that are not well understood [1–3]. Besides, OAC patients have a poor prognosis, with <20% surviving >5 years [4]. Therefore, primary prevention of OAC is an important matter in the current society. Previous and recent studies had reported that white race, male gender, obesity and tobacco smoking are all major OAC risk factors [5,6]. Several studies also reported inverse associations with consumption of fruit and vegetables for the risk of OAC [7,8].

Selenium is one of the dietary factors, which has been investigated for its possible role in cancer aetiology [9]. Evidence from laboratory and population-based studies indicate that some selenium containing compounds have anti-carcinogenic effects [10–12]. Selenium may interfere with the cancer development by inhibiting cellular proliferation, promoting apoptosis and protecting DNA from oxidative damage [13]. However, the effect of selenium on the risk of OAC is still unknown. We hypothesized that higher levels of selenium would be associated with a reduced risk of developing OAC. To investigate this hypothesis, we conducted a meta-analysis to (1) explore the relationship between selenium levels and OAC risk; (2) then evaluate the possible dose–response relationship of selenium and OAC; and (3) assess the heterogeneity among between-studies and publication bias.

## MATERIALS AND METHODS

### Search strategy

Studies were identified by using a literature search of PubMed and Web of Knowledge through June, 2015 and by handsearching the reference lists of the retrieved articles. The following search terms were used: ‘diet’ or ‘lifestyle’ or ‘selenium’ combined with ‘oesophageal adenocarcinoma’ or ‘OAC’ or ‘oesophageal carcinoma’. Two investigators (Bin Hong and Lihong Huang) searched the related articles and reviewed all the retrieved studies independently.

### Study selection

For inclusion, studies had to fulfil the following criteria: (1) prospective or case-control study design; (2) reported the relationship between selenium and OAC; (3) relative risks (RRs) or odds ratios (ORs) with 95% confidence intervals (CIs) were provided; (4) for dose–response analysis, the each category of selenium was provided (or data available to calculate them); and (5) written in English.

### Exclusion criteria

The exclusion criteria for this meta-analysis were as follows: (1) reviews; (2) the above-mentioned interests were not reported; (3) impossible to extract the appropriate data from the published results.

When the same institution reported more than once, the most recent publication was included. Two investigators carefully reviewed all identified studies independently to determine whether an individual study was eligible for inclusion criteria in this meta-analysis.

### Data extraction

Two researchers (Bin Hong and Lihong Huang) independently extracted the following data from the included studies: the first author's last name, the age for participants, publication year, geographic locations, study design, duration of follow-up, the number of cases and participants (person-years), adjustment for covariates and RRs (95% CIs) for each category of selenium. From each study, we extracted the RRs that reflected the greatest degree of control for potential confounders. Otherwise, the crude RRs with 95% CIs were extracted. Any disagreements with the two authors were resolved by discussion between the third reviewers (Liangbo Hu).

### Statistical analysis

A random-effect dose–response meta-analysis was conducted with the method proposed by Greenland and Longnecker [14] and Orsini and Bellocco [15], which takes into account the correlation between the log *RRs* estimates across categories of selenium levels. The non-linear relationships by modelling selenium levels were also explored by using restricted cubic splines with three knots at fixed percentiles (25%, 50% and 75%) of selenium levels distribution [16]. The *P*-value for non-linearity was calculated by testing against the null hypothesis that the coefficient of the second spline transformation was equal to zero [17]. The required conditions are that the number of cases and person-years or participants and the RRs (95% CIs) with the variance estimates for at least three quantitative exposure categories are known. We will estimate the slopes (linear trends) by using variance-weighted least squares regression analysis although the number of cases and person-years or participants was not available [18,19]. The median level selenium for each specific category was assigned to each corresponding log *RRs* estimate. We used the midpoint between the upper and lower boundary if the median intake was not reported in the article. If the upper boundary of the highest category was not provided, we assumed that the boundary had the same amplitude as the adjacent category. Statistical heterogeneity across studies was assessed using the *I*^2^ statistics [20], and *I*^2^ values of 0, 25, 50 and 75% represent no, low, moderate and high heterogeneity respectively [21]. Sensitivity analysis [22] was performed to describe how robust the pooled estimator risk was to removal of each individual studies. Publication bias was evaluated using Begg's funnel plot [23] and Egger's regression asymmetry test [24]. All statistical analyses were tested by STATA version 10.0. Two-tailed *P* ≤ 0.05 was accepted as statistically significant. The *P* value <0.1 was considered as significant for between-study heterogeneity and publication bias.

## RESULTS

### Literature search and study characteristics

**Figure 1:**
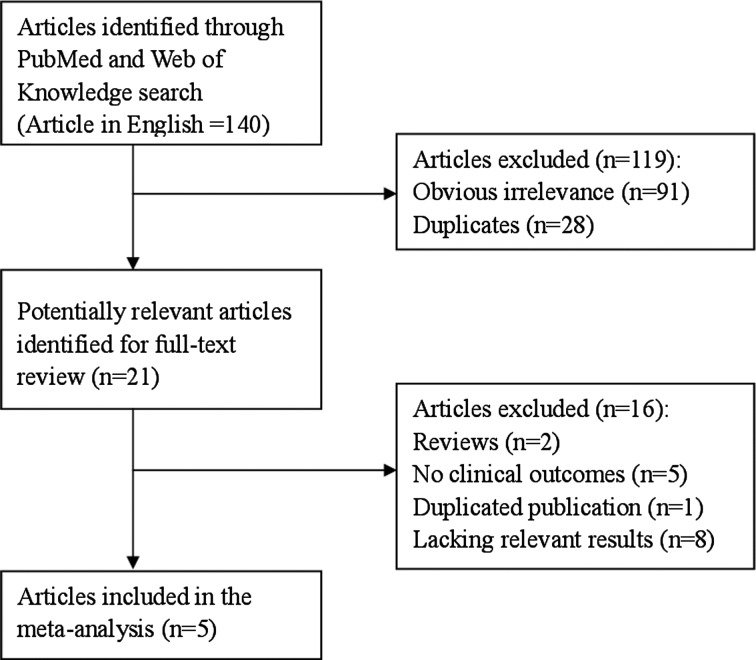
The flow diagram of screened, excluded and analyzed publications

In total, the electronic database searches 140 articles from PubMed and Web of Knowledge. After screening the title or abstract, 119 studies were excluded and 21 were retrieved and evaluated in detail. Finally, five articles [25–29] involving a total of 748 patients were included in this meta-analysis. Figure 1 presented the flow chart for exclusion/inclusion process. Five studies come from Australia, Netherland, United Kingdom and U.S.A. All included studies were retrospective or prospective studies. Among included articles, three articles were case-control design and two were cohort design. The study characteristics and participant features are given in Table 1.

**Table 1:** Characteristics of studies on selenium levels and oesophageal adenocarcinoma risk

				Age (years)				
First author (year)	Study design	Country	Participants (cases)	Case	Control	Category (μg/day)	RR (95% CI)	Adjustment for covariates
Murphy et al. (2010)	Case-control	United Kingdom	480 (224)	64±11	63±13	<5353–72≥72	11.26 (0.75–2.11)1.20 (0.72–2.00)	Adjusted for age, sex, body mass index, energy intake, smoking status, education, occupation, alcohol, regular nonsteroidal antiinflammatory drug (NSAID) use and *Helicobacter pylori* infection
Ibiebele et al. (2013)	Case-control	Australia	857 (288)	64±10	58±11	9–3737–4444–5253–165	11.08 (0.74–1.59)0.93 (0.62–1.38)1.15 (0.76–1.73)	Adjusted for gender, age, education, body mass index (BMI), oesophageal reflux symptoms, lifetime alcoholic drink, total pack-years of smoking, NSAID use, supplement use and total energy
Steevens et al. (2010)	Cohort	Netherland	7584 (64)	55–69	55–69	≤49.849.9–55.255.3–61.3>61.3	11.13 (0.67–1.91)0.84 (0.48–1.49)0.76 (0.41–1.40)	Adjusted for age, sex, cigarette smoking, frequency and duration, alcohol consumption and body mass index
O'Rorke et al. (2012)	Case-control	United Kingdom	341 (125)	64±10.3	63.6±12.7	≤61.461.5–74.6>74.6	11.03 (0.58–1.76)1.06 (0.49–2.27)	Adjusted for age at interview, sex and smoking status (current, former and never)
Takata et al. (2012)	Cohort	U.S.A.	361 (47)	64.2±10.6	61.1±11.7	<126.3 (μg/l)126.3–143.8>143.8	11.67 (0.79–3.53)1.40 (0.65–3.02)	Adjusted for age at time of blood draw, waist:hip ratio (quartiles) at baseline, sex, smoking status and NSAID use

### High compared with low analyses

**Figure 2:**
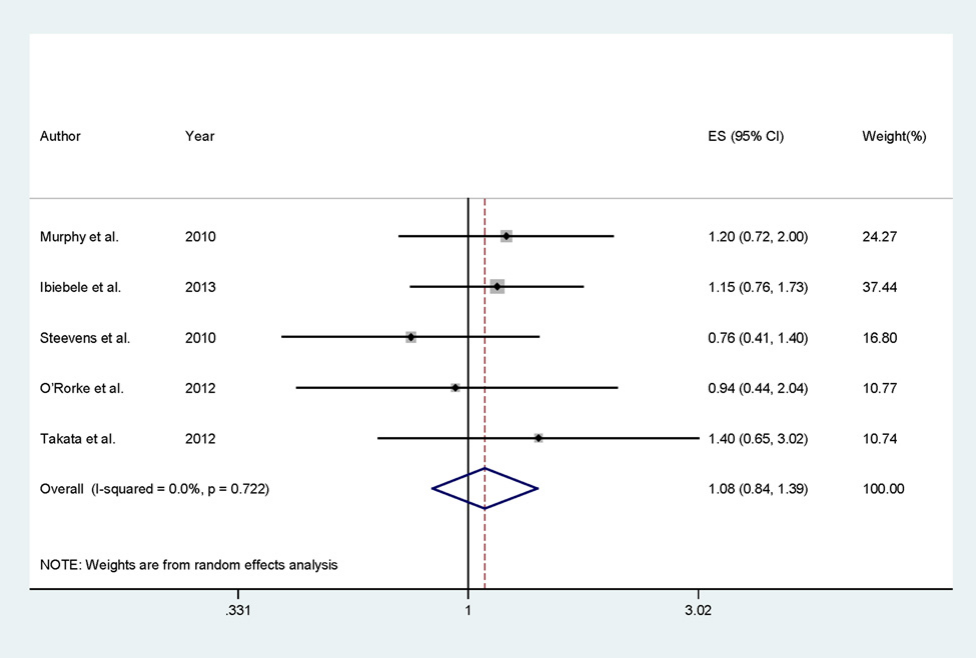
The forest plot between highest compared with lowest categories of selenium level and OAC risk

Two of the included studies reported an inverse but non-significant association between selenium levels and OAC risk, whereas three studies reported that selenium levels could increase but not significant for the OAC risk. Our pooled results suggested that the high selenium levels compared with low levels were not significantly associated with the risk of OAC (RRs=1.08, 95% CIs=0.84–1.39, *P*=0.722, *I*^2^=0%, Figure 2). When conducted subgroup analysis by study design, no significant association was found in case-control studies (RRs=1.13, 95% CIs=0.84–1.52, *P*=0.869, *I*^2^=0%) or cohort studies (RRs=0.99, 95% CIs=0.55–1.78, *P*=0.223, *I*^2^=32.6%).

### Dose–response analysis

**Figure 3:**
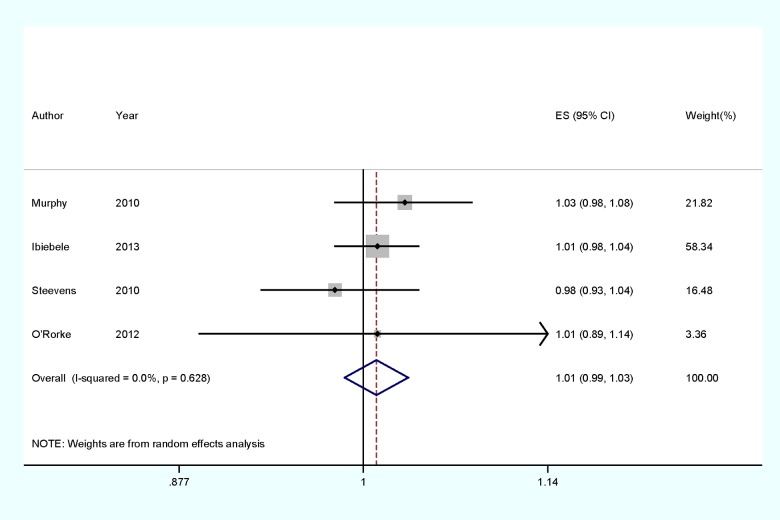
Dose–response meta-analyses of every 10 μg/day increased intake of selenium and the risk of OAC. Squares represent study-specific RRs, horizontal lines represent 95% CIs and diamonds represent summary RRs.

For dose–response analysis, data from four studies [25–28] comprising 701 OAC cases were used for selenium levels and OAC risk. Evidence of statistically significant departure from linearity (*P*_for non-linearity_=0.04) was found. Our dose–response analysis indicates that an increase in selenium intake of 10 μg/day is marginally associated with 1% increase in the risk of developing OAC (summary RRs=1.01, 95% CIs=0.99–1.03, *P*=0.424, *I^2^*=0%), but not statistically significant (Figure 3).

### Sources of heterogeneity and meta-regression

As shown in Figures 2 and 3, no evidence of heterogeneity (*I*^2^=0%, *P*_heterogeneity_=0.722) were found in the pooled results and subgroup analysis. Thus, the univariate meta-regression was not performed.

### Influence analysis and publication bias

**Figure 4:**
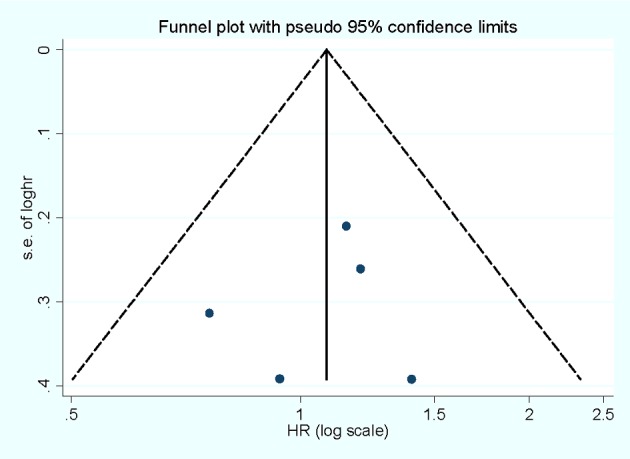
Begg's funnel plot for publication bias of selenium level and OAC risk

Influence analysis shows that no individual study exerted excessive influence on the association of selenium levels and OAC risk. Egger's test (*P*=0.738) and Begg's funnel plot (Figure 4) showed no evidence of significant publication bias was found between the association of selenium levels and OAC risk.

## DISCUSSION

The findings from this meta-analysis indicated that selenium were not significantly associated with the risk of OAC. The associations were also not significant both in case-control studies and cohort studies. An increased in selenium intake of 10 μg/day is marginally associated with 1% increase in the risk of developing OAC, but not statistically significant.

The incidence of OAC has risen dramatically in the current social. Selenium has been studied in related to the cancer risk. The reason for selenium in prevention to cancer aetiology is the antioxidant capacity of the selenium-dependent glutathione peroxidase enzymes [9,30]. Some other possible mechanisms indicated that selenium could be associated with lower cancer risk, possible because it including the reduction in inflammation, alteration of DNA methylation, induction of detoxifying phase II enzymes, induction of apoptosis of cancer cells and inhibition of angiogenesis [9]. In our study, we failed to test the hypothesis, probability due to the small number studies included in this meta-analysis.

To the best of our knowledge, this is the first comprehensive meta-analysis to explore the relationship between selenium levels and OAC risk. The dose–response analysis between selenium levels and OAC risk was also conducted. The highlights of this meta-analysis were that no between-study heterogeneity was found in the pooled analysis and subgroup analyses and large number of cases and participants were included, allowing a much greater possibility of reaching reasonable conclusions between selenium levels and OAC risk. Third, Egger's test and Begg's funnel plot showed no significant publication bias was found. However, there were some limitations in this meta-analysis should be concerned. First, our meta-analysis included three case-control studies and two cohort studies. For the case-control studies, some recall or selection bias may be inherent in the original studies. The result of the meta-regression showed that no evidence of significant between subgroup of study design, and the summary RRs were consistent in the case-control studies and cohort studies. In our results, the association was not significant either in case-control studies or in prospective studies. Therefore, more original studies especially with prospective design are wanted in the future studies. Second, other unpublished literatures on relevant pharmaceutical websites were not searched and only studies in English were included, which may lead to a potential publication bias, although no significant publication bias was found by Egger's test and Begg's funnel plot. Third, between-study heterogeneity is common in the meta-analysis, and our study found no evidence of between-study heterogeneity in the pooled analysis.

In summary, results from this meta-analysis suggested that a higher selenium level was not significantly associated with the risk of OAC. The dose–response analysis indicated that an increased in selenium intake of 10 μg/day is marginally associated with 1% increase in the risk of developing OAC, but not statistically significant. The relevant further studies are warranted.

## Abbreviations

BMI: body mass index
CI: confidence interval
NSAID: nonsteroidal antiinflammatory drug
OAC: oesophageal adenocarcinoma
RR: relative risk
